# Use of Andexanet Alfa to Reverse Heparin After Cardiopulmonary Bypass

**DOI:** 10.1016/j.atssr.2025.06.023

**Published:** 2025-07-23

**Authors:** David Niehaus, Michael Javorski, Elliot Kay, Abigail Rhoades, Hilary Raidt, Rob Dowling

**Affiliations:** 1 The Carl and Edyth Lindner Center for Research and Education at The Christ Hospital, Cincinnati, Ohio; 2 Cardiothoracic Surgery Department, Heart and Vascular Institute, The Christ Hospital, Cincinnati, Ohio; 3 Anesthesia Associates of Cincinnati, Cincinnati, Ohio; 4 Pharmaceutical Department at The Christ Hospital, Cincinnati, Ohio

## Abstract

Heparin is the primary anticoagulant used for cardiopulmonary bypass and requires reversal to prevent postoperative bleeding. Protamine sulfate is the only United States Food and Drug Administration–approved heparin reversal agent and therefore is routinely used after cardiopulmonary bypass. Severe intraoperative protamine reactions are infrequent but may preclude full heparin reversal. Andexanet alfa has demonstrated effective in vitro heparin reversal, but clinical use has not been reported. We review the preclinical data on andexanet alfa as a heparin reversal agent and report a clinical case of andexanet alfa infusion, with complete laboratory and clinical evidence of heparin reversal.

Heparin is used in most operations that require cardiopulmonary bypass (CPB). Protamine sulfate is the only United States Food and Drug Administration–approved medication to reverse the anticoagulation effect of heparin.^1^ Adverse reactions to protamine are uncommon but can be severe and may require reheparinization and reinstitution of CPB.^2^ Alternative heparin reversal strategies are not currently available. Andexanet alfa has shown promise as a reversal agent for heparin in vitro.^3^, ^4^, ^5^, ^6^ We review the preclinical data and report a successful in-human use of andexanet alfa as an effective heparin reversal agent in a patient with a severe protamine reaction.

A 61-year-old man with type 1 diabetes, biventricular failure, and recent exposure to protamine underwent a redo sternotomy for orthotopic heart transplantation. After weaning from CPB a 30-mg test dose of protamine was given. Within 1 minute of administration, there was a marked increase in both airway pressures and pulmonary artery pressures that were refractory to medical therapy. Additional heparin was given and the patient was placed back on CPB.

Owing to the severity of the protamine reaction and preclinical data on the efficacy of andexanet alpha as a heparin reversal agent,^3^, ^4^, ^5^, ^6^ a 400-mg bolus of andexanet alfa was administered over 13 minutes. The infusion was continued with an additional 480 mg at 4 mg/min. Activated clotting time (ACT) was measured before and after the initial bolus of andexanet alfa. Clinical bleeding was assessed by the attending surgeons. Chest tube drainage was measured 24 hours after surgery. Institutional Review Board approval was obtained and patient consent for publication was obtained.

The ACT value measured before andexanet alfa administration was >1005 seconds. ACT values measured 4 minutes and 10 minutes after andexanet alfa administration were 161 seconds and 156 seconds, respectively, to achieve partial heparin reversal. This was due to concerns for a potential hypercoagulable state leading to thrombosis. Clinically, there was a marked reduction in bleeding consistent with complete heparin reversal. Chest tube output was 108 mL during the first 24 hours postoperative. The patient had an uneventful recovery and was discharged home 11 days after his operation.

## Comment

Heparin is the primary anticoagulant for maintaining therapeutic anticoagulation while on CPB. Protamine is the only United States Food and Drug Administration–approved heparin reversal agent. Alternative strategies, such as a universal heparin reversal agent and ciraparantag (PER977) for heparin reversal, have been extensively studied preclinically but lack significant clinical data and are not approved for use.^4^ The incidence of anaphylactic reactions due to protamine allergic reactions ranges from 0.06% to 10.6%.^7^ Patients with a history of protamine exposure, vasectomy, fish allergies, and insulin-use diabetes are at increased risk of an adverse reaction.^8^

Andexanet alfa is a recombinant modified inactivated human factor Xa decoy protein, developed for reversal of both direct and indirect factor Xa inhibitors.^6^ Preclinical in vitro data demonstrated that andexanet produced dose-dependent neutralization of heparin’s effects on anti-Xa and IIa activity, activated partial thromboplastin time, ACT, thrombin time, and thromboelastography variables.^3^, ^4^, ^5^, ^6^ Although andexanet alfa has shown heparin reversal in vitro, andexanet alfa has not been used as a heparin reversal agent in a clinical setting.

A potential downside for using this drug is financial, with the hospital cost in this case being $12,250. The cost of this drug may be partially offset by decreased need for blood products with their attendant costs. Another concern is creation of a hypercoagulable state, which is attempted to be avoided by only partially correcting the coagulation variables.

In conclusion, preclinical data were used to support the use of andexanet alfa as a reversal agent in a patient with a severe protamine reaction. Laboratory data and clinical observation demonstrated success with andexanet alfa as a heparin reversal agent. Owing to the current cost, use of this drug will be limited to treatment of patients with the most severe protamine reactions. Further clinical experience may demonstrate that andexanet alfa can achieve rapid reversal of heparin in select cases with an acceptable safety profile when protamine is contraindicated.
